# AI-Assisted Passive Magnetic Distance/Position Sensor

**DOI:** 10.3390/s25041132

**Published:** 2025-02-13

**Authors:** Chaoyi Qiu, Zhenghong Qian, Qiao Qi, Ruigang Wang, Xiumei Li, Ru Bai

**Affiliations:** 1 School of Information Science and Technology, Hangzhou Normal University, Hangzhou 311121, China; qiucy@stu.hznu.edu.cn (C.Q.); qiqiao@hznu.edu.cn (Q.Q.); ruigangwang@hznu.edu.cn (R.W.); xiumei_li@hotmail.com (X.L.); bairu@hdu.edu.cn (R.B.); 2 Center for Integrated Spintronic Devices, Hangzhou Dianzi University, Hangzhou 310018, China

**Keywords:** magnetic sensing, nonlinear magnetic field, distance/position sensor, BP neural network

## Abstract

Magnetic sensing technology is crucial for non-contact distance and position measurement. Due to the nonlinear characteristics of the magnetic fields from permanent magnets, conventional magnetic sensors struggle with accurate distance and position determination. To address this, we propose a distance/position sensor that employs a customized back propagation (BP) neural network. By detecting the magnetic field variations induced by a permanent magnet, the proposed sensor can effectively model the nonlinear mapping between magnetic field strength and distance, thereby enabling precise distance and position measurement. Experimental results demonstrate that the BP neural network approach, when employing a single magnetic sensor, exhibits a measurement error in the range of −0.0268 mm to 0.0362 mm over a distance of 0–70 mm, which is significantly lower than traditional methods based on the magnetic dipole model and the Levenberg–Marquardt (LM) algorithm. Increasing the number of sensors to three reduces the error further to −0.0107 mm to 0.0093 mm. Furthermore, when employing four magnetic sensors for position measurement within a 60 mm × 60 mm planar area, the positioning errors along the *x*-axis and *y*-axis are confined to the ranges of −0.6168 mm to 1.1312 mm and −0.6001 mm to 0.5133 mm, respectively.

## 1. Introduction

Distance and position measurement using magnetic fields is a technique that employs magnetic sensors to assess the relative distance or position of a magnetic target. This is achieved by analyzing variations in magnetic field intensity and orientation induced by the target at various spatial locations. Magnetic targets can be broadly categorized into two types: permanent magnets [1,2] and electromagnetic coils [3,4]. Among them, permanent magnets do not necessitate an external power source to sustain their magnetic field, making them highly suitable for wireless distance measurement and position measurement applications.

Compared to other distance measurement and position measurement techniques, such as potentiometer-based distance measurement [5], strain gauge-based distance measurement [6], laser-based distance measurement [7], ultrasonic distance measurement and position measurement [8], and vision-based position measurement [9], magnetic field-based distance measurement and position measurement boasts several advantages, including high precision, high sensitivity, non-contact nature, wear resistance, low cost, and immunity to line-of-sight obstructions [10]. As a result, this technology has found widespread application across diverse fields, including precision control of industrial machinery, liquid level monitoring, vibration assessment, thickness measurement, indoor localization [11], and capsule endoscopy [12].

Significant progress has been made in the field of magnetometry for determining the distance and position of magnetic targets. Balevičius et al. in [13] developed a magnetic proximity sensor that incorporates a cylindrical permanent magnet and a scalar magnetic field sensor. Their research demonstrated that this sensor effectively detects distances to both ferromagnetic and non-magnetic metallic objects. However, since it measures only the scalar magnetic flux density, it cannot determine the specific direction. Tsai et al. in [14] developed a non-contact distance measurement device based on the giant magnetoresistance (GMR) effect. In their design, a neodymium magnet is mounted on a tool slide that moves in conjunction with it, resulting in a variation in the distance between the neodymium magnet and the GMR sensor. The distance of the slide can be calculated by non-contact measurement of magnetic field strength using the GMR sensor. Within an effective measurement range of 500 μm, the error is approximately ±0.65 μm. However, this limited effective measurement range restricts its application in most scenarios. Nara et al. [15] introduced a simplified algorithm for linear position measurement that utilizes magnetic dipoles to calculate the magnetic gradient tensor. This method enables localization of magnetic targets without the need to consider the orientation of the magnetic dipole, relying solely on the three components of the magnetic field and the magnetic gradient tensor measured at a single measurement site. The maximum error is roughly 7 mm when there is an 80–140 mm gap between the sensor and the magnetic object. Although the calculation is fast, the accuracy is relatively low. Schlageter et al. [16] developed a permanent magnet position measurement system that consists of a 16-sensor Hall array and estimates the permanent magnet’s five-dimensional (5-D) posture using an LM optimization algorithm. This system achieves a measurement range of up to 140 mm, with an average tracking error of 3 mm and an angular error of 1.2 degrees, albeit at the cost of system complexity due to the requirement of a 4 × 4 sensor array, leading to relatively larger errors. Furthermore, the starting settings of the posture parameters have a significant impact on the LM algorithm’s tracking accuracy and computational efficiency, making it prone to local minima and resulting in long computation times, especially when the initial values deviate significantly from the true values, leading to potential convergence issues. Thus, some studies have applied AI to magnetic localization to address this issue. References [17,18,19] present magnetic localization methods that utilize AI-based methods to provide initial solutions for traditional optimization algorithms; this can effectively enhance the performance of magnetic positioning; however, such enhancement relies on the performance of the traditional algorithms themselves. In addition, Su S et al. in [20] propose a model-driven AI magnetic positioning algorithm, but its applicability is limited by specific mathematical models. Unlike the aforementioned literature, this study employs a data-driven AI magnetic localization algorithm that is not constrained by specific mathematical models. Additionally, the data used includes random measurement errors, and the final results are directly derived from the neural network, free from the limitations of traditional optimization algorithms, thereby offering broader applicability.

In fact, many applications, such as level measurement and precision control of industrial machinery, require not only the detection of the proximity of a magnetic target to a magnetic sensor but also, given the known orientation of the magnetic target, the precise determination of the relative distance or planar position between the magnetic target and the sensor. Traditional algorithms for magnetic distance and position measurement depend on optimizing high-order nonlinear equations obtained from a model of a magnetic dipole, inverting the magnetic target’s pose using the LM algorithm. Yet, the real link between the magnetic field and the magnetic target’s posture is approximated by the magnetic dipole model. Significant inaccuracies may arise from this, especially if there is fewer than three times the target’s size between the magnetic target and the measuring site. Motivated by these limitations, we propose a method that employs a backpropagation (BP) neural network to fit the real magnetic field information and the distance and planar position of a magnetic target. This method is trained on data to capture the complex relationship between the magnetic field and the location of the magnetic target, resulting in enhanced accuracy.

The remainder of this article is structured as follows: Section 2 outlines the methods used for measuring the distance and planar position of a magnetic target, providing a brief overview of the magnetic dipole theory and experimental system structure; Section 3 details the experimental setup and presents the results; while Section 4 summarizes and discusses the backpropagation neural network algorithm, as well as future work directions for measuring the distance and planar position of magnetic targets.

## 2. Methods

### 2.1. Magnetic Dipoles and Measurement System

In this paper, we take an axially magnetized cylindrical magnet as the magnetic target and employ an array of triaxial magnetic sensors to measure the magnetic target’s location and distance. When the size of the magnetic target is less than three times the distance to the measuring point, the magnetic dipole can precisely simulate the magnetic field produced by the magnet. In this scenario, for computational reasons, the magnetic object can be efficiently handled as a magnetic dipole [21]. By employing magnetic sensors or sensor arrays and modeling magnetic targets as dipoles, the relative distance between the target and the center of the sensor array can be ascertained.

**Figure 1:**
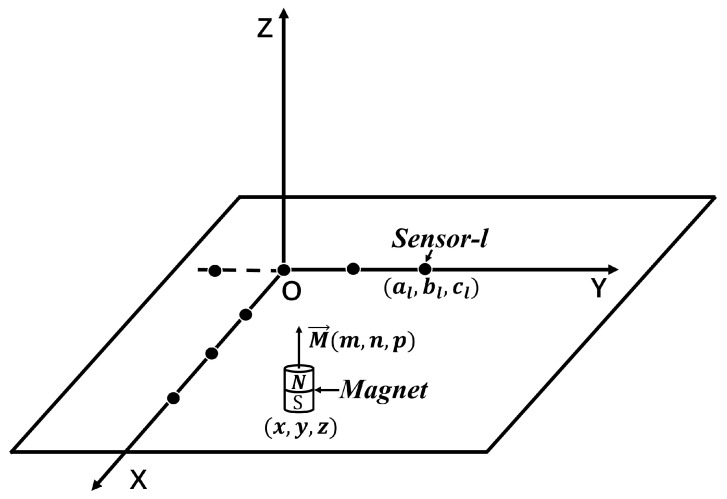
Location of magnetic targets and sensors.

Without loss of generality, we design a system for distance and 2D position measurement, as shown in Figure 1, where the magnetic target and *N* sensors are arranged, allowing for the determination of the target’s distance and planar location based on magnetic field data acquired by the sensors. In the distance measurement experiments, black solid circles representing sensors are distributed on the *y*-axis, while the magnetic target moves on the positive *x*-axis. The magnetic target moves inside the *x*-*y* plane, while sensors are positioned along the positive *x*-axis for planar positioning studies.

Let $\vec{M}\left(m,n,p\right)$ be the magnetic dipole moment vector and the center coordinates of the magnetic target be (x,y,z). The location of the *l*-th sensor is $\left(a_{l},b_{l},c_{l}\right)$ and the unit vector pointing from the target (x,y,z) to the sensor $\left(a_{l},b_{l},c_{l}\right)$ is $\vec{r_{l}}$, with its magnitude being $|R_{l}|=\sqrt{{\left(a_{l}-x\right)}^{2}+{\left(b_{l}-y\right)}^{2}+{\left(c_{l}-z\right)}^{2}}$. The unit vectors $\vec{i},\vec{j},\vec{k}$ correspond to the x,y and *z*-axes, respectively. Then, the three-axis magnetic induction intensity $\vec{B_{l}}$ measured by the *l*-th sensor due to the magnetic target can be expressed as:

(1) $$\vec{B_{l}}=B_{lx}\vec{i}+B_{ly}\vec{j}+B_{lz}\vec{k}=B_{T}\left(\frac{3\left(\vec{M}\;\cdot \;\vec{r_{l}}\right)\vec{r_{l}}}{|R_{l}{|}^{5}}-\frac{\vec{M}}{|R_{l}{|}^{3}}\right),$$

where $B_{T}=\frac{\mu_{0}}{4\pi }$ is a constant determined by the properties of the material with given $\mu_{0}=4\pi \times 10^{-7}$ T·m/A as the permeability of free space. Expanding Equation (1) with respect to $B_{lx},B_{ly}$ and $B_{lz}$ yields:

(2) $$\begin{array}{c} B_{lx}=B_{T}\left\{\frac{3\left[m\left(a_{l}-x\right)+n\left(b_{l}-y\right)+p\left(c_{l}-z\right)\right]\left(a_{l}-x\right)}{R_{l}^{5}}-\frac{m}{R_{l}^{3}}\right\}\\ B_{ly}=B_{T}\left\{\frac{3\left[m\left(a_{l}-x\right)+n\left(b_{l}-y\right)+p\left(c_{l}-z\right)\right]\left(b_{l}-y\right)}{R_{l}^{5}}-\frac{n}{R_{l}^{3}}\right\}.\\ B_{lz}=B_{T}\left\{\frac{3\left[m\left(a_{l}-x\right)+n\left(b_{l}-y\right)+p\left(c_{l}-z\right)\right]\left(c_{l}-z\right)}{R_{l}^{5}}-\frac{p}{R_{l}^{3}}\right\} \end{array}$$

To simplify the experiment, the magnetic moment is fixed along the (0,0,1) direction, and both the magnetic object and the sensor array are maintained at a constant height, with their plane parallel to the *x*-*y* plane. As a result, the direction vector pointing to the *l*-th sensor from the magnetic target position can be simplified to $\vec{r_{l}}=\left(a_{l}-x,b_{l}-y,0\right)$, and the three-axis magnetic induction intensity measurements obtained by the sensor can be expressed as:

(3) $$B_{lz}=-B_{T}\frac{p}{R_{l}^{3}}.$$

By substituting the z-coordinate of the magnetic target, its magnetic moment, and the positions of the sensor array into Equation (1) and performing an inverse calculation, it is possible to reverse the magnetic target’s location. The functional relationship between the location of the magnetic target (x,y) and the magnetic induction intensity $\vec{B_{l}}$ can be stated as:

(4) $$\left(x,y\right)=f^{-1}\left(\vec{B_{l}}\right).$$

Given the high-order nonlinear characteristics of the magnetic dipole model [22], the measurement of the magnetic target’s position can be framed as an optimization problem involving high-order nonlinear equations. Therefore, the function based on the magnetic dipole can be solved using nonlinear optimization algorithms [23]. The following is the expression for the error function, which measures the discrepancy between the theoretical values produced from the magnetic dipole model and the magnetic field data acquired from the sensor:

(5) $$E=\sum_{l=1}^{N}\left|\right|\vec{B_{ml}}-\vec{B_{l}}{\left|\right|}^{2}.$$

where $\vec{B_{ml}}=\left(B_{mlx},B_{mly},B_{mlz}\right)$ represents the measured magnetic field information from the *l*-th magnetic sensor, and $\vec{B_{l}}=\left(B_{lx},B_{ly},B_{lz}\right)$ denotes the matching theoretical value that was determined using the magnetic dipole model.

Based on Equations (4) and (5), to reduce the error and precisely estimate the magnetic target’s pose, nonlinear optimization algorithms might be used. Newton’s method [24], the Gauss-Newton method [25], and the conjugate gradient method [26] are typical nonlinear optimization algorithms, while the LM algorithm [27] is most commonly used for optimizing magnetic dipole-based functions due to its high accuracy and speed. However, the initial value of the LM algorithm has a significant impact on its precision and efficacy. The technique may converge to a local minimum if the initial estimate differs greatly from the true value. This could lead to a drop in efficiency or even the algorithm’s inability to converge at all. Additionally, when the distance between the magnetic target and the measurement point is less than three times the size of the magnetic target, the magnetic dipole model introduces significant errors. Therefore, neural networks can be employed to model the nonlinear relationship between the actual magnetic field data and the distance or location of the magnetic target, thereby addressing the errors caused by the magnetic dipole model and transforming the magnetic dipole optimization problem into a regression problem [28]. To sum up, a BP neural network is employed for fitting, and its results are compared with those of applying the LM method to optimize the magnetic dipole model.

### 2.2. Customized BP Neural Network

A BP neural network is a multi-layer feedforward artificial neural network (ANNs) that makes use of the error backpropagation algorithm. It is known that the BP neural network is renowned for its ability to approximate complex nonlinear functions [29]. The theoretical foundation of the BP neural network lies in its ability to minimize the error between predicted outputs and actual outputs through iterative training. This process allows the network to adapt and learn from the data, improving the accuracy of its predictions over time [30]. In our research, the magnetic field generated by the permanent magnet exhibits significant nonlinearity, which poses challenges for traditional measurement techniques. By leveraging the BP neural network’s capability to learn and model the nonlinear relationship between magnetic field strength and distance, we were able to achieve more precise measurements.

**Figure 2:**
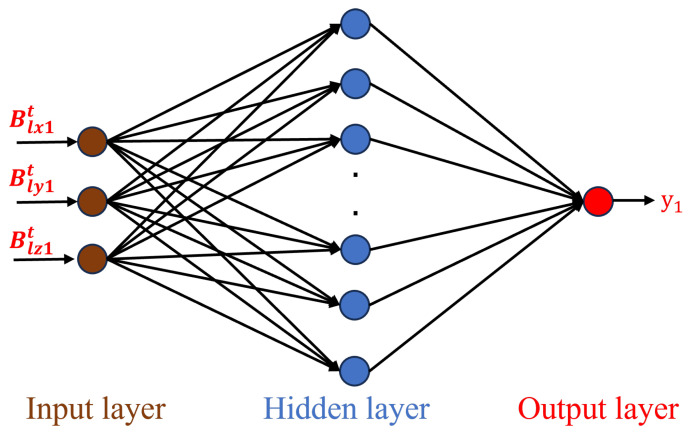
BP neural network for distance measurement.

**Figure 3:**
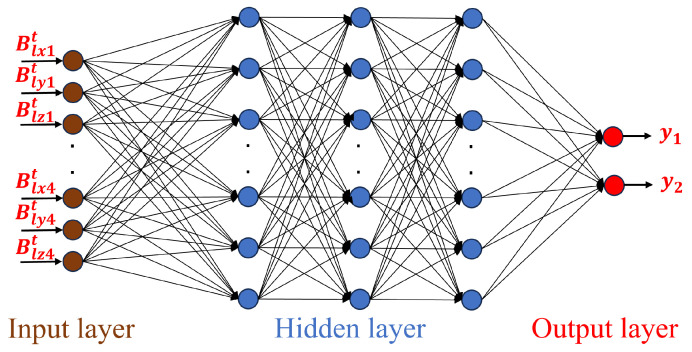
BP neural network for position measurement.

An input layer, a hidden layer, and an output layer make up the majority of a BP neural network model’s architecture. Neurons in adjacent layers are fully connected, but not those in the same layer or between non-adjacent layers. As illustrated in Figure 2 and Figure 3, we design and train BP neural networks for distance measurement and planar position measurement, respectively.

The data for distance measurement, denoted as $\left\{\left(X_{1D}^{t},Y_{1D}^{t}\right)|t=1∼N\right\}$, are defined as the training dataset, where *N* is the quantity of training samples, $X_{1D}^{t}={\left[B_{1x}^{t},B_{1y}^{t},B_{1z}^{t}\right]}^{T}$ is the input vector, with $B_{1x}^{t},B_{1y}^{t},B_{1z}^{t}$ denoting the magnetic induction intensity components measured along the *x*, *y* and *z*-axes by a single sensor, respectively, and $Y_{1D}^{t}={\left[y_{1}^{t}\right]}^{T}$ is the output vector, with $y_{1}^{t}$ representing the distance corresponding to the measured magnetic induction intensity. As shown in Figure 2, a BP neural network with m hidden layer nodes is defined for distance measurement as follows:

(6) $$\sum_{i=1}^{m}V_{i}f\left(W_{i}X_{1D}^{t}-\theta_{i}\right)=Y_{1D}^{t},$$

where $W_{i}={\left[w_{1i},w_{2i},w_{3i}\right]}^{T}$ is the connection weights between the input neuron and the *i*-th hidden layer neuron; $V_{i}={\left[v_{i1}\right]}^{T}$ is the connection weights between the *i*-th hidden layer neuron and the output layer neuron; the bias of the *i*-th hidden layer neuron is denoted by $\theta_{i}$; the hidden layer’s activation function is denoted by f(·).

The data for planar position measurement, denoted as $\left\{\left(X_{2D}^{t},Y_{2D}^{t}\right)|t=1∼N\right\}$, are defined as the training dataset, where *N* is the number of training samples, $X_{2D}^{t}={\left[B_{1x}^{t},B_{1y}^{t},B_{1z}^{t},\ldots ,B_{4x}^{t},B_{4y}^{t},B_{4z}^{t}\right]}^{T}$ is the input vector with $B_{1x}^{t},B_{1y}^{t},B_{1z}^{t},\ldots ,B_{4x}^{t},B_{4y}^{t},B_{4z}^{t}$ representing the *x*,*y*, and *z* components of the magnetic induction intensity measured by four sensors, respectively, and $Y_{2D}^{t}={\left[y_{1}^{t},y_{2}^{t}\right]}^{T}$ is the output vector with $y_{1}^{t},y_{2}^{t}$ representing the *x* and *y* coordinates, respectively, corresponding to the measured magnetic induction intensity. As shown in Figure 3, a BP neural network consisting of 3 hidden layers and the number of nodes are *m*, *n*, and *l* respectively is defined for distance measurement as follows:

$$\sum_{k=1}^{l}O_{k}^{\prime }f\left(\sum_{j=1}^{n}H_{j}^{\prime }f\left(\sum_{i=1}^{m}V_{i}^{\prime }f\left(W_{i}^{\prime }X_{2D}^{t}-\theta_{i}\right)-\theta_{j}\right)-\theta_{k}\right)=Y_{2D}^{t},$$

where $W_{i}^{\prime }={\left[w_{1i},w_{2i},\ldots ,w_{12i}\right]}^{T}$ is the connection weights between the input neuron and the *i*-th neuron of the first hidden layer; $V_{i}^{\prime }={\left[v_{i1},v_{i2},\ldots ,v_{in}\right]}^{T}$ is the connection weights between the *i*-th neuron of the first hidden layer and the second hidden layer; $H_{j}^{\prime }={\left[h_{j1},h_{j2},\ldots ,h_{jl}\right]}^{T}$ is the connection weights between the *j*-th neuron of the second hidden layer and the third hidden layer; $O_{k}^{\prime }={\left[o_{k1},o_{k2}\right]}^{T}$ is the connection weights between the *k*-th neuron of the third hidden layer and the output layer; the biases are denoted by $\theta_{i}$, $\theta_{j}$, $\theta_{k}$; the hidden layer’s activation function is denoted by f(·).

For the selection of training algorithms for BPNNs, the Levenberg–Marquardt (LM) algorithm, widely employed in data fitting applications, represents a hybrid approach combining the gradient descent and Gauss–Newton (GN) methods. By concurrently leveraging both first and second-order derivative information, LM offers a robust optimization solution. Although each iteration of the LM algorithm is computationally more expensive, its exploitation of the mean squared error as the objective function allows for a clever avoidance of the Hessian matrix, thereby mitigating the computational burden through increased efficiency [31]. Furthermore, in numerous instances where conjugate gradient (CG) and variable learning rate algorithms fail to converge, the LM algorithm proves to be successful [32]. Consequently, the LM algorithm is chosen to train the BPNN in this study. Consequently, the LM algorithm is chosen to train the BPNN in this study.

These networks can fit real magnetic fields and learn the relationship between the magnetic field and the target’s distance and planar position, enabling high-precision and computationally efficient distance and planar position measurement of magnetic targets. To assess the accuracy of distance and planar position measurements, we introduce two key error metrics: distance error and planar position error. Distance error is defined as the discrepancy between the measured distance and its true value, as shown in Equations (7) and (8), whereas planar position measurement error represents the deviation between the measured planar position and its corresponding true position, as shown in Equations (9) and (10).

(7) $$MAE_{1D}=\frac{1}{n}\sum_{i=1}^{n}\left|a_{m}-a_{e}\right|,$$

(8) $$RMSE_{1D}=\frac{1}{n}\sum_{i=1}^{n}\sqrt{{\left(a_{m}-a_{e}\right)}^{2}},$$

(9) $$MAE_{2D}=\frac{1}{n}\sum_{i=1}^{n}\left(|a_{m}-a_{e}|+|b_{m}-b_{e}|\right),$$

(10) $$RMSE_{2D}=\frac{1}{n}\sum_{i=1}^{n}\sqrt{{\left(a_{m}-a_{e}\right)}^{2}+{\left(b_{m}-b_{e}\right)}^{2}},$$

where *n* denotes the quantity of samples in the testing set, $a_{m}$ and $b_{m}$ denote the true measured *x* and *y* coordinates of the magnetic target, respectively, $a_{e}$ and $b_{e}$ represent the predicted *x* and *y* coordinates of the magnetic target obtained from the BP neural network.

## 3. Experimental Results

This experiment consists of two components. The first component primarily investigates the impact of different sensor numbers and algorithms on distance measurement, specifically including distance measurement experiments using a single sensor with a BP neural network (BPNN) algorithm, distance measurement experiments using a magnetic dipole-based algorithm, and distance measurement experiments using three sensors with a BPNN algorithm, followed by a comparative analysis of the experimental findings. In the second part, the effectiveness of a BPNN algorithm using four sensors in a planar positioning task is assessed.

### 3.1. Dataset

Given the requirement for a substantial amount of training data in neural network training, a finite element simulation model of the magnetic target is initially constructed using Ansys Electronics Desktop 2021 R1 to generate a substantial dataset for analyzing the correlation between the relative distance or planar position between the sensor measurement sites and the magnetic target, and the magnetic induction intensity measured at the sensor measurement points.

In the pre-processing stage of finite element modeling, the material properties, including a definition of the magnetic target as an N52 grade NdFeB magnet with a remanence Br of 1.45 T, a coercivity Hc of 860 kA/m, and axial magnetization, as well as the establishment of the geometric model (a cylinder measuring 8 mm in diameter and 8 mm in height) and boundary conditions, are completed.

**Figure 4:**
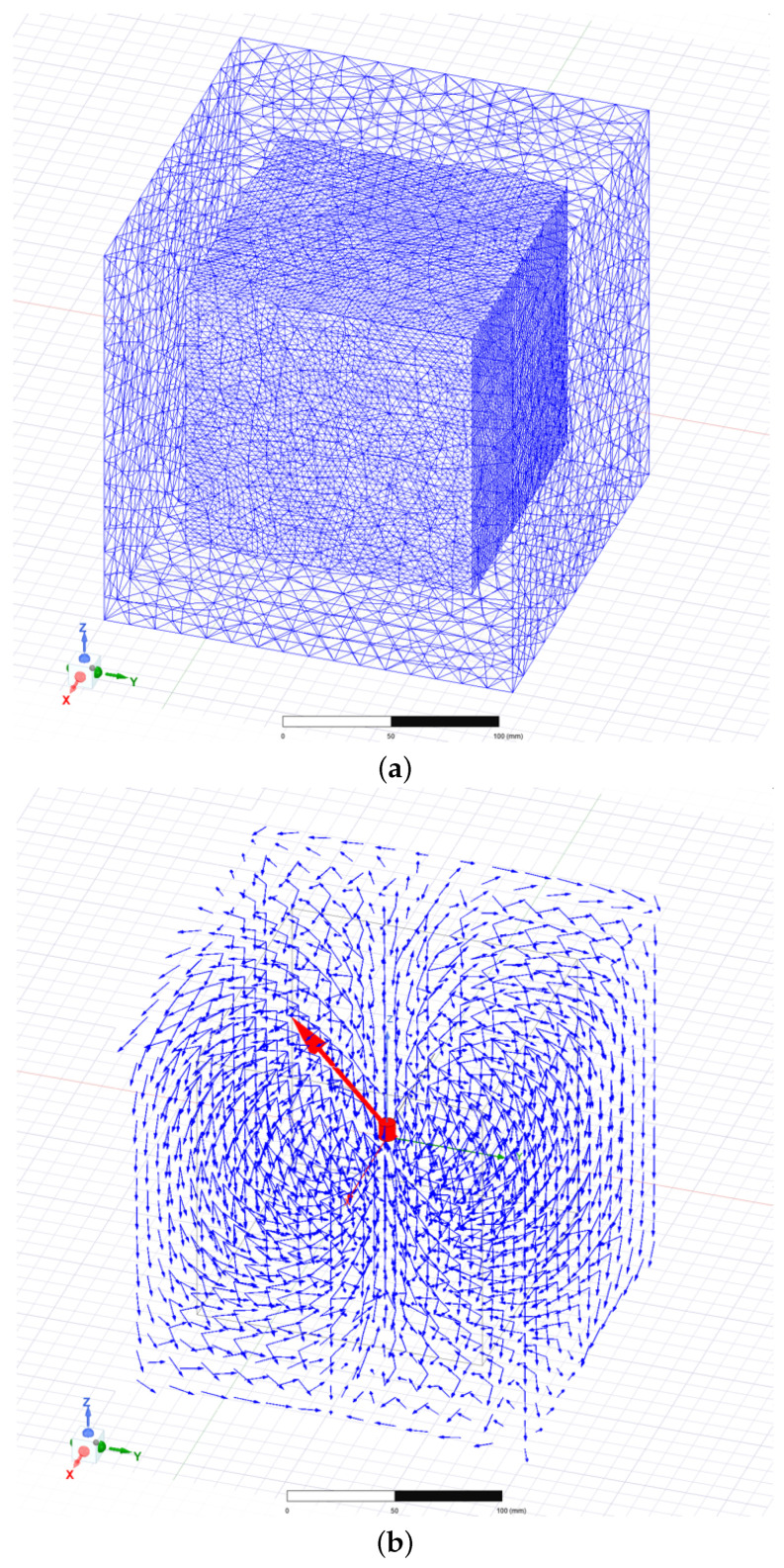
(**a**) Finite Element Mesh of the Magnetic Target; (**b**) Magnetic Induction Intensity Distribution of the Magnetic Target.

A meshing scheme is employed, wherein a maximum element size of 20 mm is specified within the region bounded by (−100 mm, −100 mm, −100 mm) and (100 mm, 100 mm, 100 mm), while a finer mesh with a maximum element size of 5 mm is defined within the subregion bounded by (−70 mm, −70 mm, −70 mm) and (70 mm, 70 mm, 70 mm). Leveraging the adaptive meshing capabilities of Ansys Electronics Desktop 2021 R1, the mesh is iteratively refined to minimize errors, converging after two iterations with a final tetrahedral element count of 1,130,044. The resulting mesh and magnetic induction vector field are illustrated in Figure 4a.

In post-processing, the simulated magnetic induction vector field of the magnetic target obtained from the simulation experiment is shown in Figure 4b. By extracting data from the simulation, two datasets are created, respectively, for training neural networks for distance measurement and planar position measurement.

To construct the distance measurement dataset, 3 sensors are evenly distributed along the *y*-axis with center coordinates of (0 mm, −10 mm, 4 mm), (0 mm, 0 mm, 4 mm), and (0 mm, 10 mm, 4 mm), respectively. The magnetic target moved along the positive *x*-axis within a range of 0 mm to 70 mm, with coordinates from (4 mm, 0 mm, 4 mm) to (70 mm, 0 mm, 4 mm) and a minimum movement interval of 0.01 mm. A total of 6601 data points are collected, each containing the distances between the 3 sensors and the magnetic target, as well as the 9 magnetic induction intensity vector components measured by the 3 sensors. To construct the planar positioning dataset, 4 sensors are evenly distributed along the *y*-axis with center coordinates of (0 mm, 0 mm, 4 mm), (0 mm, 20 mm, 4 mm), (0 mm, 40 mm, 4 mm), and (0 mm, 60 mm, 4 mm), respectively. The magnetic target moved within a 60 mm × 60 mm area on the *x*-*y* plane, with coordinates ranging from (0 mm, 0 mm, 4 mm) to (60 mm, 60 mm, 4 mm) and a minimum movement interval of 0.5 mm. A total of 14,614 data points are collected, each containing the planar coordinates of the 4 sensors and the corresponding 12 magnetic induction intensity vector components measured by the 4 sensors.

### 3.2. 1D Distance Measurement Based on BPNN

Both a single sensor and an array of 3 sensors are employed to conduct distance measurement experiments using backpropagation neural networks.

In the distance measurement experiment with a single sensor, the 3 magnetic induction intensity vector components of the sensor located at (0 mm, 0 mm, 4 mm) are used as input, while the distance between this sensor and the magnetic target is used as output. A subset of the simulated data, denoted as $\left\{\left(X_{1D}^{t},Y_{1D}^{t}\right)|t=1∼N\right\}$, is defined as the training dataset, where *N* is the quantity of training samples, $X_{1D}^{t}={\left[B_{1x}^{t},B_{1y}^{t},B_{1z}^{t}\right]}^{T}$ is the input vector with $B_{1x}^{t},B_{1y}^{t},B_{1z}^{t}$ denoting the magnetic induction intensity components measured along the *x*, *y* and *z*-axes by a single sensor, respectively, and $Y_{1D}^{t}={\left[y_{1}^{t}\right]}^{T}$ is the output vector with $y_{1}^{t}$ representing the distance corresponding to the measured magnetic induction intensity. The neural network with a single hidden layer is trained using the Levenberg-Marquardt algorithm.

In the distance measurement experiment using a 3-sensor array, all 9 features collected from the 3 sensors in the entire dataset are used as input, while the distance between the magnetic target and the sensors serves as the output. The entire dataset, denoted as $\left\{\left(X_{1D\prime }^{t},Y_{1D\prime }^{t}\right)|t=1∼N\right\}$, is defined as the training dataset, where *N* is the quantity of training samples, $X_{1D\prime }^{t}={\left[B_{1x}^{t},B_{1y}^{t},B_{1z}^{t},B_{2x}^{t},B_{2y}^{t},B_{2z}^{t},B_{3x}^{t},B_{3y}^{t},B_{3z}^{t}\right]}^{T}$ is the input vector with $B_{1x}^{t},B_{1y}^{t},B_{1z}^{t},B_{2x}^{t},B_{2y}^{t},B_{2z}^{t},B_{3x}^{t},B_{3y}^{t}$, and $B_{3z}^{t}$ represents the *x*, *y* and *z* components of the magnetic induction intensity measured by three sensors, respectively. $Y_{1D\prime }^{t}={\left[y_{1}^{t}\right]}^{T}$ is the output vector, with $y_{1}^{t}$ representing the distance corresponding to the measured magnetic induction intensity. A single-hidden-layer neural network is trained using the Levenberg–Marquardt algorithm.

**Figure 5:**
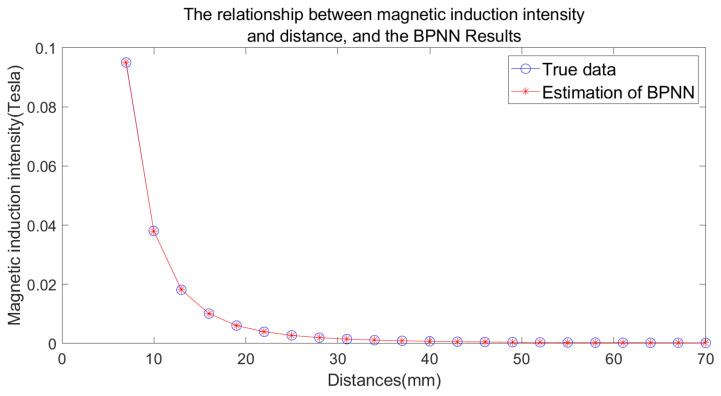
The relationship between magnetic induction intensity and distance and a comparison of the actual and predicted distances from the BPNN in the test set when using a single sensor.

Figure 5 illustrates the nonlinear relationship between magnetic induction intensity and distance (shown by blue circle), with the horizontal axis representing the output distance and the vertical axis representing the scalar value of the magnetic induction intensity. However, a backpropagation neural network can effectively fit this nonlinear relationship, establishing a one-to-one correspondence between magnetic induction intensity and distance. The distance to the magnetic target can be calculated by measuring the magnetic induction intensity at a certain location. Also, Figure 5 shows a comparison between the actual and predicted distances (indicated by a red star) in the test set when using a single sensor, demonstrating the network’s ability to fit the relationship between these two variables. The relationship between magnetic induction intensity and distance is nonlinear, and the distance to be calculated is a function of the variable magnetic induction intensity. After fitting the relationship between magnetic induction intensity and distance using a BPNN, the corresponding distance can be obtained by measuring the magnetic induction intensity, making the distance measured by the sensor linearly related to the actual moving distance of the sensor.

**Figure 6:**
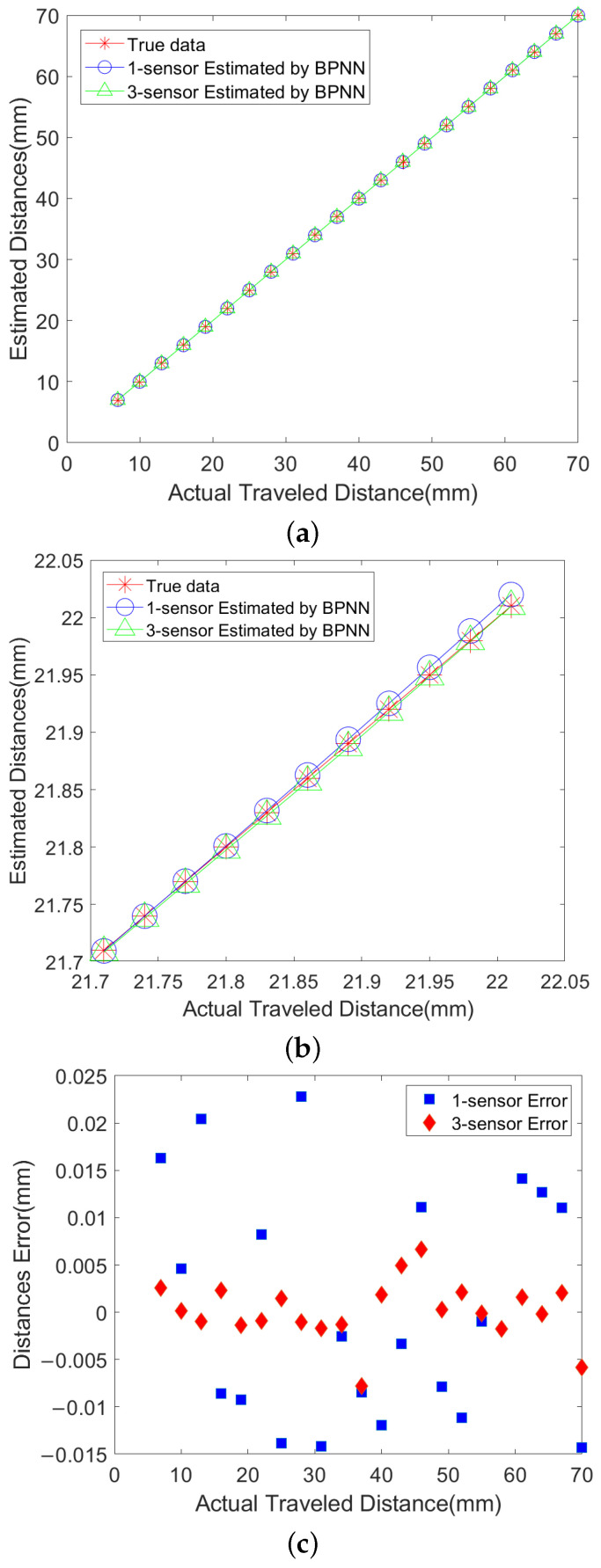
(**a**) Diagram of Measured Distance and Actual Travel Distance; (**b**) A magnified local comparison of measured distance versus actual travel distance; (**c**) Diagram of Distance Errors for Test Samples.

Figure 6a,b shows a comparison of the predicted values and actual values of the distance in the BPNN test set. In contrast, Figure 6c presents the corresponding distance measurement error results. In order to more clearly compare the effects of a single sensor and three sensors on the distance measurement accuracy, the comparison diagram in the range of 21.7 mm–22.05 mm is enlarged. In both figures, the horizontal axis indicates the actual moving distance of the sensor, while the vertical axis reflects the distance measured by the sensor, in units of mm. In the comparison plot of predicted and actual values from the BPNN, red stars represent the true distance between the magnetic sensor and the magnetic target, blue circles represent the expected distance between a single sensor and the magnetic target, and green triangles represent the expected distance between three sensors and the magnetic target. In the distance measurement error result plot of the BPNN, blue squares represent the error between a single sensor and the magnetic target, and red diamonds represent the error between three sensors and the magnetic target. Furthermore, the distance measurement error on the entire test set using a single sensor is in the range of −0.0268 mm to 0.0362 mm, with a $MAE_{1D}$ of 0.0091 mm and a $RMSE_{1D}$ of 0.0114 mm. When using three sensors, the distance measurement error of the BPNN method is reduced to a range from −0.0107 mm to 0.0093 mm on the entire test set, with a $MAE_{1D}$ of 0.0022 mm and an $RMSE_{1D}$ of 0.0028 mm.

The real data collected by sensors often contain random measurement errors. Therefore, we incorporated random measurement errors into the simulated data and conducted comparative experiments using BPNN for distance measurements.

Table 1 and Table 2 present the results of distance measurements using one and three sensors, respectively, after adding random measurement errors. The first column shows the different levels of random measurement errors added, with a mean of 0 and ranges was 0, −0.1 to 0.1, −0.2 to 0.2, −0.3 to 0.3, −0.4 to 0.4, −0.5 to 0.5, and −1 to 1 μT. The second, third, and fourth columns represent the range of localization error, $MAE_{1D}$, and $RMSE_{1D}$, respectively, all in units of mm. Experimental results show that as the random measurement error increases from 0 to ±1 μT, the range of localization error of distance measurement using one sensor increases from −0.0268 mm~0.0362 mm to −0.3366 mm~0.2726 mm, the $MAE_{1D}$ increases from 0.0091 mm to 0.0471 mm, and the $RMSE_{1D}$ increases from 0.0114 mm to 0.0664 mm. For distance measurements using three sensors, the range of localization error increases from −0.0107 mm~0.0093 mm to −0.1639 mm~0.1661 mm, the $MAE_{1D}$ increases from 0.0022 mm to 0.0146 mm, and the $RMSE_{1D}$ increases from 0.0028 mm to 0.0264 mm. This means that even after adding larger random measurement errors, the distance measurement accuracy using three sensors is still higher than that using one sensor, but all the indicators of distance measurement decrease with the increase of random measurement errors.

### 3.3. Comparison of Distance Measurement Accuracy Between the BPNN and the Magnetic Dipole-Based Algorithm

**Figure 7:**
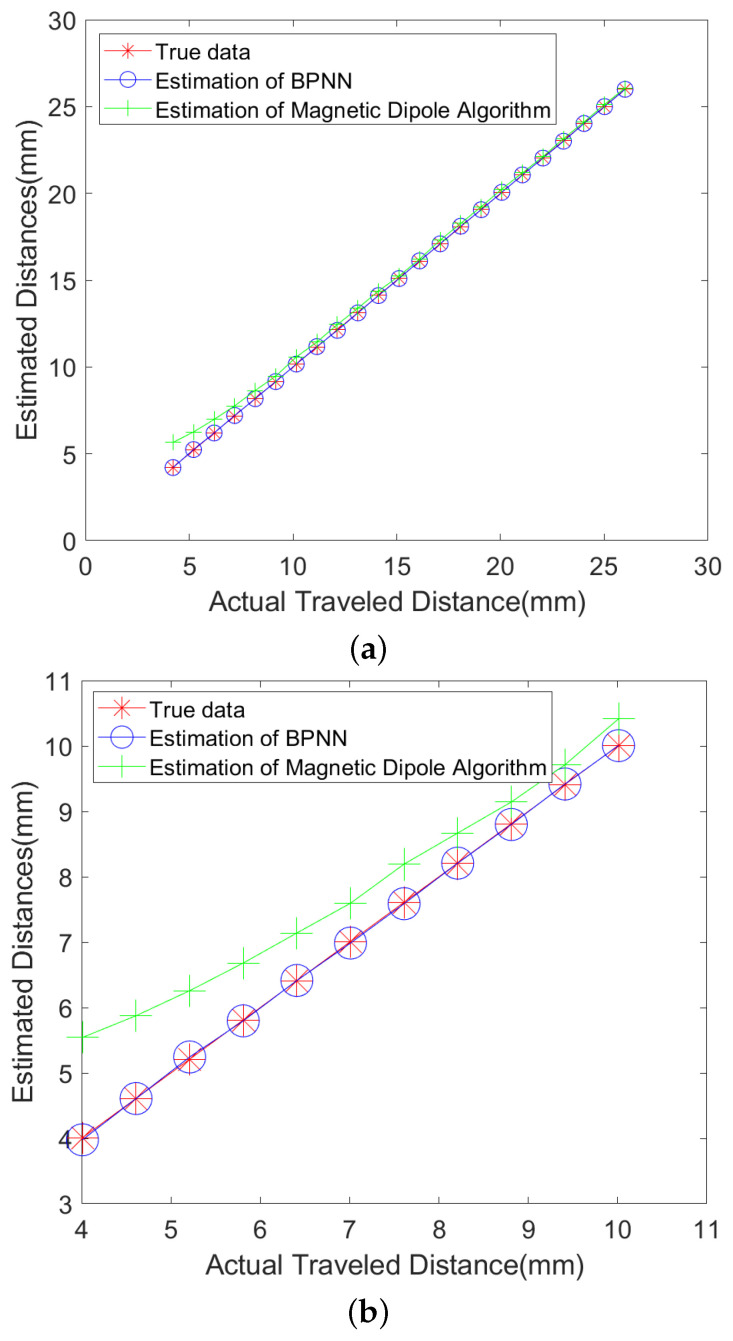
(**a**) BPNN Results and Magnetic Dipole Algorithm Results for Test Samples; (**b**) Magnified local comparisons of BPNN and magnetic dipole algorithm results for test samples.

The magnetic dipole model may produce large inaccuracies if the distance between the magnetic target and the measuring site is less than three times the target’s size. Therefore, when the distance is small, optimization of the magnetic dipole-based function using the LM algorithm results in large errors. In contrast, the BPNN effectively addresses this issue. Since the magnetic source used in our experiments has a diameter and height of 8 mm, we compared the distance measurement results obtained using BPNNs and applying the LM method to optimize the magnetic dipole model in the range of 4 mm to 24 mm. Figure 7a,b shows the predicted distances using both methods.

The horizontal axis represents the actual distance traveled by the sensor in millimeters, while the vertical axis indicates the measured distance obtained from the sensor also in millimeters. Red stars indicate the expected distance between the magnetic sensor and target, blue circles represent the distances predicted by the BPNN, and green plus signs represent the distances predicted by applying the LM method to optimize the magnetic dipole model. As shown in the figure, applying the LM method to optimize the magnetic dipole model exhibits larger errors when the distance between the magnetic target and measurement point is small. Within the 4 mm to 5 mm range, the BPNN achieves $MAE_{1D}$ of 0.0111 mm and $RMSE_{1D}$ of 0.0199 mm, while the optimizing the magnetic dipole model using the LM algorithm has $MAE_{1D}$ of 1.327 mm and an $RMSE_{1D}$ of 1.326 mm. These results illustrate that the BPNN outperforms applying the LM method to optimize the magnetic dipole model in terms of distance measurement accuracy, especially when the sensor and magnetic target are in close proximity.

To conduct a deeper comparison between the BPNN and the LM algorithm, multiple BPNNs were trained with varying numbers of samples within the same experimental range as the previous study.

As shown in Table 3, the performance of BPNN trained with different numbers of samples was evaluated. As the number of training data points decreased from 6601 to 1651, the range of localization error of BPNN increased from −0.0268~0.0362 mm to −1.2141 mm~0.4595 mm, the $MAE_{1D}$ increased from 0.0091 mm to 0.1383 mm, and the $RMSE_{1D}$ increased from 0.0114 mm to 0.1917 mm. When the number of training data points decreased to 826, the range of localization error of BPNN further increased to −20.3987 mm~3.1187 mm, the $MAE_{1D}$ increased to 1.3233 mm, and the $RMSE_{1D}$ increased to 1.7708 mm. It is clear that the positioning accuracy of BPNN decreases significantly with the decrease in the number of training samples. When the sample size is 1651, all indicators are an order of magnitude lower than those when the sample size is 6601 and are on the same order of magnitude as the results obtained by optimizing the magnetic dipole-based function using the LM algorithm. This means that only about 1651 data points are needed to achieve a positioning accuracy comparable to that obtained by optimizing the magnetic dipole-based function using the LM algorithm.

### 3.4. 2D Position Measurement on BPNNs

**Figure 8:**
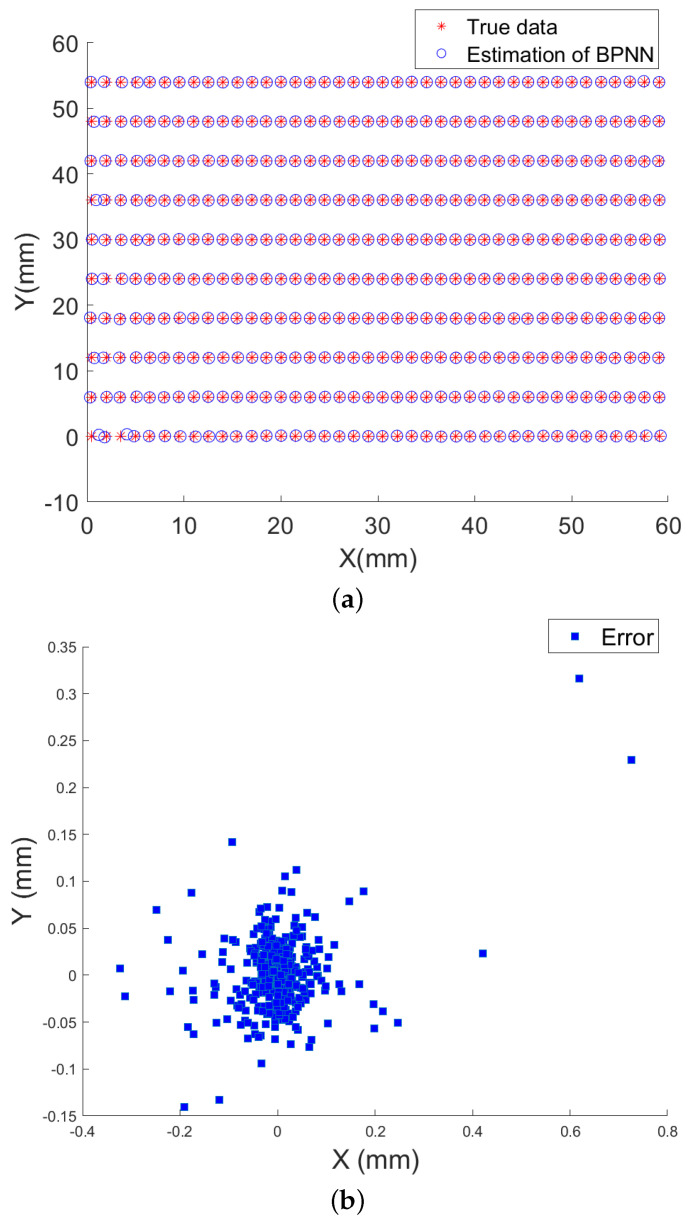
(**a**) Diagram of Measured Distance and Actual Travel Distance; (**b**) Diagram of Distance Errors for Test Samples.

Similar to the previous experiments, the application of BPNN can be extended from distance measurement to planar position measurement. Figure 8a presents a comparison between the predicted and actual planar positions obtained using the BPNN. In addition, Figure 8b presents a plot of the planar position measurement errors.

When training the BPNN for planar location measurement, the 12 components of the magnetic induction vector in the corresponding dataset are used as input, and the relative planar position coordinates between the magnetic sensor and target are used as output. A subset of the simulated data $\left\{\left(X_{2D}^{t},Y_{2D}^{t}\right)|t=1∼N\right\}$ is defined as the training dataset, where *N* is the number of training samples, $X_{2D}^{t}={\left[B_{1x}^{t},B_{1y}^{t},B_{1z}^{t},\ldots ,B_{4x}^{t},B_{4y}^{t},B_{4z}^{t}\right]}^{T}$ is the input vector with $B_{1x}^{t},B_{1y}^{t},B_{1z}^{t},\ldots ,B_{4x}^{t},B_{4y}^{t},B_{4z}^{t}$ representing the *x*, *y*, and *z* components of the magnetic induction intensity measured by four sensors, respectively, and $Y_{2D}^{t}={\left[y_{1}^{t},y_{2}^{t}\right]}^{T}$ is the output vector with $y_{1}^{t},y_{2}^{t}$ representing the *x* and *y* coordinates, respectively, corresponding to the measured magnetic induction intensity. Since this neural network requires fitting more parameters compared to the BPNN used for distance measurement, to better learn more complex functional relationships and improve generalization ability, the neural network is configured with three hidden layers, utilizing the Levenberg–Marquardt algorithm as the training algorithm.

In Figure 8a,b, the vertical axis shows the associated *y*-coordinate, while the horizontal axis shows the matching *x*-coordinate of the measurement location, both in millimeters. In the True Plane Positions and BPNN Results in the Test Set plot, blue solid circles denote the expected relative positions between the magnetic sensor and target, while red solid circles represent the predicted relative positions. In the Plane Positions Errors plot, blue squares indicate the error between the expected and predicted relative positions. Furthermore, the network achieves the *x*-axis positioning error within a range of −0.6168 mm to 1.1312 mm, with $MAE_{2D}$ of 0.0414 mm and $RMSE_{2D}$ of 0.0811 mm, and the *y*-axis positioning error is within a range of −0.6001 mm to 0.5133 mm, with $MAE_{2D}$ of 0.0257 mm and $RMSE_{2D}$ of 0.0364 mm over the entire test set.

The real data collected by sensors often contain random measurement errors. Therefore, we incorporated random measurement errors into the simulated data and conducted comparative experiments using BPNN for localization measurements.

Table 4 and Table 5 present the results of *x*-axis and *y*-axis localization measurements using BPNN, respectively, after adding random measurement errors. The first column indicates the different levels of random measurement errors added, with a mean of 0 and ranges of 0, −0.1 to 0.1, −0.2 to 0.2, −0.3 to 0.3, −0.4 to 0.4, −0.5 to 0.5, and −1 to 1 μT. The second, third, and fourth columns represent the range of localization error, $MAE_{2D}$, and $RMSE_{2D}$, respectively, all in units of mm. Experimental results show that as the random measurement error increases from 0 to ±1 μT, the *x*-axis range of localization error increases from −0.6168 mm ~1.1312 mm to −5.3168 mm ~3.4234 mm, the $MAE_{2D}$ increases from 0.0414 mm to 0.1119 mm, and the $RMSE_{2D}$ increases from 0.0811 mm to 0.2143 mm. Similarly, the *y*-axis range of localization error increases from −0.6001 mm~0.5133 mm to −4.7766 mm~4.4347 mm, the $MAE_{2D}$ increases from 0.0257 mm to 0.0901 mm, and the $RMSE_{2D}$ increases from 0.0364 mm to 0.2275 mm. Among them, $MAE_{2D}$ and $RMSE_{2D}$ represent the overall accuracy of the trained model, and they increase with the rise of random measurement error, indicating that the accuracy of the trained model declines as the random measurement error increases.

## 4. Conclusions

Based on a BPNN, we present an enhanced passive magnetic distance/position sensor in this research. Firstly, we conducted finite element simulations using Ansys to collect a dataset. The magnetic induction intensity from the collected data was used as the input, while the distance and position were used as the output. After that, the BPNN was trained using this dataset. Experimental results demonstrate that when using a single magnetic sensor, the measurement error obtained by the BPNN approach falls between −0.0268 mm and 0.0362 mm within a linear range of 0–70 mm, significantly outperforming the conventional magnetic distance measurement method based on magnetic dipoles and the LM algorithm. Further research shows that increasing the number of sensors can further improve measurement accuracy. When the quantity of magnetic sensors is increased to three, the measurement error of the BPNN method within the same linear range is reduced to a range from −0.0107 mm to 0.0093 mm. Additionally, this paper successfully achieves millimeter-level two-dimensional planar positioning using four magnetic sensors. Experiments conducted within a 60 mm × 60 mm planar range show that the *x*-axis positioning error is within a range of −0.6168 mm to 1.1312 mm, and the *y*-axis positioning error is within a range of −0.6001 mm to 0.5133 mm. Therefore, the proposed method exhibits superior performance in both distance measurement and positioning. Furthermore, we are currently establishing a real experimental platform and will conduct real experiments to confirm this work in future research.

## Figures and Tables

**Table 1:** Comparison of the impact of different measurement errors on BPNNs used for distance measurement (1-sensor).

Measurement Errors (μT)	Range of Localization Error (mm)	${\mathrm{MAE}}_{1D}$ (mm)	${\mathrm{RMSE}}_{1D}$ (mm)
0	−0.0268~0.0362	0.0091	0.0114
−0.1~0.1	−0.0421~0.0449	0.0108	0.0135
−0.2~0.2	−0.1359~0.0829	0.0174	0.0236
−0.3~0.3	−0.1437~0.0952	0.0184	0.0243
−0.4~0.4	−0.1360~0.1068	0.0203	0.0271
−0.5~0.5	−0.2063~0.1448	0.0245	0.0342
−1~1	−0.3366~0.2726	0.0471	0.0664

**Table 2:** Comparison of the impact of different measurement errors on BPNNs used for distance measurement (3-sensors).

Measurement Errors (μT)	Range of Localization Error (mm)	${\mathrm{MAE}}_{1D}$ (mm)	${\mathrm{RMSE}}_{1D}$ (mm)
0	−0.0107~0.0093	0.0022	0.0028
−0.1~0.1	−0.0194~0.0244	0.0033	0.0049
−0.2~0.2	−0.0349~0.0413	0.0057	0.0085
−0.3~0.3	−0.0501~0.0655	0.0061	0.0099
−0.4~0.4	−0.0579~0.0709	0.0077	0.0126
−0.5~0.5	−0.0722~0.0868	0.0085	0.0146
−1~1	−0.1639~0.1661	0.0146	0.0264

**Table 3:** Comparison of distance measurement results based on different training sample sizes.

The Number of Training Data Points	Range of Localization Error (mm)	${\mathrm{MAE}}_{1D}$ (mm)	${\mathrm{RMSE}}_{1D}$ (mm)
6601	−0.0268~0.0362	0.0091	0.0114
3301	−0.0439~0.0421	0.0122	0.0153
2201	−0.0498~0.0474	0.0431	0.0572
1651	−1.2141~0.4595	0.1383	0.1917
826	−20.3987~3.1187	1.3233	1.7708

**Table 4:** Comparison of the impact of different measurement errors on BPNNs used for position measurement (*x*-axis).

Measurement Errors (μT)	Range of Localization Error (mm)	${\mathrm{MAE}}_{2D}$ (mm)	${\mathrm{RMSE}}_{2D}$ (mm)
0	−0.6168~1.1312	0.0414	0.0811
−0.1~0.1	−0.8633~1.1539	0.0526	0.1026
−0.2~0.2	−1.0743~1.5910	0.0608	0.1159
−0.3~0.3	−2.3165~2.2873	0.0643	0.1256
−0.4~0.4	−1.7075~4.1441	0.0832	0.1566
−0.5~0.5	−1.8325~3.3648	0.0835	0.1663
−1~1	−5.3168~3.4234	0.1119	0.2143

**Table 5:** Comparison of the impact of different measurement errors on BPNNs used for position measurement (*y*-axis).

Measurement Errors (μT)	Range of Localization Error (mm)	${\mathrm{MAE}}_{2D}$ (mm)	${\mathrm{RMSE}}_{2D}$ (mm)
0	−0.6001~0.5133	0.0257	0.0364
−0.1~0.1	−0.8623~0.7415	0.0338	0.0513
−0.2~0.2	−0.9995~1.7519	0.0367	0.0604
−0.3~0.3	−0.8928~0.8799	0.0541	0.0822
−0.4~0.4	−2.3141~2.4506	0.0676	0.1221
−0.5~0.5	−2.9282~3.3441	0.0727	0.1396
−1~1	−4.7766~4.4347	0.0901	0.2275

## Data Availability

The data presented in this study are available on request from the corresponding author.
